# The tell-tale heart: heart rate fluctuations index objective and subjective events during a game of chess

**DOI:** 10.3389/fnhum.2012.00273

**Published:** 2012-10-08

**Authors:** María J. Leone, Agustín Petroni, Diego Fernandez Slezak, Mariano Sigman

**Affiliations:** ^1^Physics Department, School of Sciences, University of Buenos AiresBuenos Aires, Argentina; ^2^Computer Science Department, School of Sciences, University of Buenos AiresBuenos Aires, Argentina

**Keywords:** decision-making, cognitive processes, problem solving, heart rate, chess, planning, calculation

## Abstract

During a decision-making process, the body changes. These somatic changes have been related to specific cognitive events and also have been postulated to assist decision-making indexing possible outcomes of different options. We used chess to analyze heart rate (HR) modulations on specific cognitive events. In a chess game, players have a limited time-budget to make about 40 moves (decisions) that can be objectively evaluated and retrospectively assigned to specific subjectively perceived events, such as setting a goal and the process to reach a known goal. We show that HR signals events: it predicts the conception of a plan, the concrete analysis of variations or the likelihood to blunder by fluctuations before to the move, and it reflects reactions, such as a blunder made by the opponent, by fluctuations subsequent to the move. Our data demonstrate that even if HR constitutes a relatively broad marker integrating a myriad of physiological variables, its dynamic is rich enough to reveal relevant episodes of inner thought.

## Introduction

The decision-making process is accompanied by modification in heart rate (HR). In the period before making a decision, HR and skin conductance changes have been related to specific cognitive events and to the load of mental work (Bradley, 2009; Jennings et al., 2009; Taelman et al., 2011); they also have been postulated to act as signals of the possible future outcomes of a decision (Damasio, 1994). After the decision, body changes have been related to the outcome of the decision (Crone et al., 2004).

One specific theory, the somatic marker hypothesis (SMH), postulates that body signals guide decision making in uncertain situations (Damasio, 1994). According to this theory, the appearance of a specific body state associated with a specific outcome previously learnt is hypothesized to signal the expected value of a choice (Tranel et al., 1999). This provides physiological evidence for what has been popularly referred as “hunches” or “gut-feelings” which provide a rapid approximate evaluation of a complex problem (Bechara and Damasio, 2005; Dijksterhuis et al., 2006; Ariely, 2008). However, this theory has been contested and there is heated controversy about the specific relation and causality of body signals and decision making (Dunn et al., 2006).

One of the most widely used setups to investigate the interaction of emotions, decision making and body signals is the Iowa Gambling Task (IGT), a card game where subjects have to choose between four decks to maximize their money gain (Bechara et al., 1994). Changes in skin conductance and HR have been showed to predict performance on the IGT (Bechara et al., 1997; Crone et al., 2003; Drucaroff et al., 2011) even before this knowledge is expressed as conscious rational thought (Bechara et al., 1997). However, the necessity of conscious knowledge has been questioned (Maia and McClelland, 2004).

Compared to the IGT and other simple decision-making protocols used in laboratory setups, the game of chess constitutes a very rich and quantitative model of real life decisions, with a virtually infinite number of states and paths. First, players make successive decisions (around 40 each) on a finite time-budget and every decision (move) can be accurately evaluated with current algorithms. Second, player expertise level can be accurately assessed (Elo, 1978; Van Der Maas and Wagenmakers, 2005). Third, players can recognize specific events of their inner thinking during the game (like planning, calculation, and error moments), which explains why chess has been a goldmine for studies of introspection (De Groot, 1965). Last but not least, this game is played in a social setup in which the relation to the other opponent sets a regulatory focus which governs the type of play (Slezak and Sigman, 2011) and constitutes a highly motivating setup. Throughout the game, players undergo strong emotional fluctuations.

Here we have studied HR variation as a physiological correlate of decision making using rapid chess as a natural experimental setup. All subjects were expert chess players, and rating differences between players were minimized to avoid opponent level-related effects (Slezak and Sigman, 2011). This time budget (15 min per player) is set as a compromise to generate move durations which are fast enough to investigate transitions in HR but also sufficiently slow to allow a player to retrospectively recall relevant moments perceived and experimented during the game. Our aim was to investigate which aspects of HR index objective variables (the quality of a move, determined by the change in the objective evaluation of the position) and subjective reports such as the conception of a plan or a moment of calculus, as reported by the player in an after game recollection of its inner thought.

## Materials and methods

### Participants

Twenty-five games were played by nine different-subjects (one to five games each). Twenty-five independent games were played, 19 by men and 6 by women, mean age 35.6 ± 11.7 years old (age range: 21–58), mean international rating (Elo) 2111 ± 60.4 (Elo range: 2021–2216). Eight games were played in a special tournament with electronic chess boards and clocks (DGT), and the rest were played using a computer. For experimented players, there should be not differences between these modalities.

### Experiment design

In all games we recorded 2 min of rest before and after the games (except the tournament games for which we recorded rest only after the game). Each chess game lasted at most 30 min.

After the final rest, players were asked to complete a meta-cognitive questionnaire (Appendix). Players reported moments in which they were engaged in establishing a plan (planning) and moments in which they were engaged with depth search, examining and evaluating concrete tactical variations (calculation). Here we use the chess convention, where planning refers to the process of setting a goal, a strategic and general aim (De Groot, 1965; Kotov, 1971). In General Problem Solving, planning often refers to explicit examination of the process to reach a known goal, i.e., the evaluation of a tree of variations, which here, as in chess, is called calculation. They filled the form including the specific move in chess algebraic notation only on those fields that they could recognize and remember from the game, not from a retrospective evaluation of the position.

### Data acquisition and preproccesing

Electrocardiogram (ECG) activity was recorded using two external electrodes on a Biosemi Active-Two system (Biosemi, Amsterdam, Holland) with a sample rate of 256 Hz (electrode location: one on the left chest and the other on the sternum). ECG data were filtered between 1 and 50 Hz, and after a global visual inspection a threshold was set to detect peaks on the ECG signal (R peaks). Signal was then converted in instantaneous HR by interpolation and referred to the mean HR of each game.

Computer games were played using JinChess (http://www.jinchess.com/), an open-source chess client which connects to a server for playing chess through Internet (FICS, Free Internet Chess Server, http://www.freechess.org/). To control network lag, we used JinChess with timeseal, a program that act as a relay station and keeps track of transmission times. To synchronize the games with the ECG signal, we modified the JinChess code, to register and save all relevant tags of the game. This signal was sent to the Active-Two system through the parallel port, identifying each event with a different 16-bit code.

### Chess data

#### Time variables

For each move we recorded player and opponent available times (AT), and the time it takes to make the move, defined for consistency with psychological experiments as response time (RT). We also defined the time after move (TAM) as the time between a move and the following opponent move. For all games, AT started in 900 s (15 min) and decreased during player's turn to play. In chess, each player has its own clock which stops during the other player's turn. If a player uses all his/her AT, the game is over (player lost by time). When AT gets close to a few seconds players have to play very fast, a situation referred as time trouble.

#### Score (S)

Score is a measure of the value of the position in pawns units. It can be seen as an estimate of the likelihood of the final result. We used the Rybka 4 engine to calculate chess moves score, using a 12 movements depth (Sigman et al., 2010). *S* > 0 indicates a white player advantage and *S* < 0, black player advantage. Score was saturated in +10 and −10. For simplicity and consistency of data presentation, we calculated a player corrected score whose sign indicates the goodness of the recorded player's position: positive values when he/she had advantage, independently on if he/she is playing with white or black pieces.

#### Delta score (ΔS)

The change in the position value (score, not player corrected) is a measure of the move goodness defined as Δ *S* = [*S* (*i* + 1) − *S* (*i*)] × *C*, where *C* is –1 for black moves and +1 for white ones. As with the score, *C* is just a correction variable to measure Δ*S* relative to the player independently of piece colors. Close to or zero values of Δ*S* indicate that player made a good move. Significant negative departures of Δ*S* from zero indicate that the player move was far from the best. We defined moves with Δ*S* ≤ −1 as blunders (errors or bad moves). Δ*S* > 0 values indicate that the player made a move that was better than all the ones conceived by the engine. Since we use an engine much stronger than all our players, this is very infrequent (see Sigman et al., 2010).

#### Phases

Chess games were classified in three conventional phases: Opening, Middle game, and Endgame by author (MJL) who is a Woman International Master (WIM). Phases were determined according to the piece distribution in the board. For instance, opening was finished when piece development was completed (not according to theorical knowledge). Although the precise transition between two phases (for instance the end of the opening and beginning of middle game) might be controversial, none of the analysis reported here is sensitive to slight changes in this criterion.

#### Move statistics

Two thousand and eighty-six moves were obtained from the 25 games (Opening: 565, Middle game: 1007, Endgame: 514). Eight hundred and sixteen of these moves had RT and TAM ≥5 s. Blunders (Δ*S* ≤ −1) were 153 (recorded players: 68; opponents: 85). Players identified 26 planning and 41 calculation moves across all games (8 moves were highlighted as both planning and calculation).

### HR dynamics

We analyzed HR dynamics around moves in a 10 s time-window centered in the move. The baseline for each move was defined between 5 and 3 s before the move, and subtracted. We used a strict criterion to avoid wrapping artifacts and contamination by move overlapping, considering only those moves with RT and TAM of at least 5 s.

#### Matching

To analyze the effect of a move category (player blunder, opponent blunder, planning, and calculation) on HR, we matched other variables, to assure that the results were not accounted by covariations in the data. For instance, as the game proceeds, players have less AT, start playing faster and are more prone to make errors.

For every category with a small number of exemplar moves we found a matched category in the complementary group with other variables matched. For example, to investigate the effect of planning we first considered all moves where subjects reported a plan. This group of moves was much smaller than its complement which assured that in principle we could find sufficient non-planning moves with the same properties in other variables (AT, Score, etc.). If matching could not be made accurately, we only considered a subset of planning moves which could be adequately matched, through a random replacement procedure. Matching conditions were determined allowing a maximal difference between each exemplar move and its match in other variables. For player versus opponent moves, matched variables were as follows: player and opponent AT (<30 s), score (<1), and Δ *S* (<0.5). Planning and calculation moves were matched on player AT (<30 s), score (<1) and Δ*S* (<0.5). All these moves were also not blunder moves. For blunder versus non-blunder moves (both player and opponent) matching variables were player AT (<30 s) and score (<1).

The resulting number of moves for each category which could be matched for all other variables was as follows: player blunders *N* = 24, opponent blunder *N* = 34, planning *N* = 15, calculation *N* = 17.

### Linear classifier analysis

We trained a support vector machine (SVM) algorithm (Cristianini and Shawe-Taylor, 2000) to test if HR could be used to classify a move as a target (a move defined by a category, as a planning or calculation move) or a non-target move (for each group target-matched moves) using a leave-four-out procedure. We used 300 independent iterations by randomly selecting the four exemplars not used in training and left for classification. For robustness of this procedure, we run the classifier five times with different matched moves for each target group.

### Statistical analysis

Correlation analysis was assessed using Pearson correlation test. Analysis of HR dynamics was carried out using Wilcoxon rank sum test comparing two groups. For each pair of conditions, we considered significantly different if *p* < 0.05 and if the point is part of a cluster of 64 points (250 ms time window).

## Results

### HR variations throughout the game

**Figure 1:**
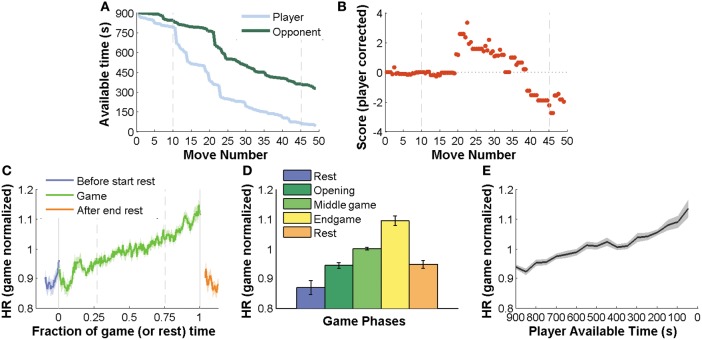
**HR correlates with game state variables.** Main chess variables: player and opponent available time (AT), game phase and score. Game advance is represented by move number. For each move number there are two movements: one of the player and other of the opponent. **(A)** Player and opponent AT throughout a typical game. AT starts at 900 s (the budget for all the games in this experiment were 15 min per player) and decreases continuously. First it decreases slowly (opening moves are played fast) and as players devote more time to each move, AT decreases more rapidly. **(B)** Score progression throughout a typical game. Score is a measure of the value of each position. Positive or negative values indicate which player had advantage: positive values when the recorded player is better and negative for opponent advantage. **(C)** HR variations above the mean averaged for all games of the experiment. Before and after the game, we measured HR at rest. **(D)** Mean HR on both rest periods and game stages (opening, middle game, and endgame) for all games of the experiment. **(E)** HR as a function of AT averaged across all games. Vertical lines on panels **(A)** and **(B)** indicate stage transitions for this specific game. On panels **(C)**, vertical solid lines indicate rest to game transitions and vertical dashed lines represent the average time for each game stage transition.

The evolution of chess-variables during a game followed an expected path, AT decreased from its initial budget of 900 s first slowly (opening moves are played fast) and in the middle game with sharp transitions revealing long moments of thought (Figure 1A). Score begun equal and showed moderate fluctuation in the opening stage. As the game proceeded, the likelihood of making an error increases due to shortage of time and complexity of the position, revealing larger fluctuations in score (Figure 1B).

HR increased steadily throughout the game (Figure 1C, average slope of HR versus fraction of game played: 6.90*e*−04 ± 3.18*e*−04, One-sample *t*-test, *p* < 0.00001), with its categorical equivalent, through the three stages of the game [One-Way ANOVA, *F*_(4, 113)_ = 27.37, *p* < 0.0001] (Figure 1D) and with AT (Figure 1E, *r* = −0.6347, *p* < 0.00001). This effect was very robust, every single game of the 25 studied here showed a negative linear correlation with AT (game slopes: −3.88*e* −04 ± 2.05*e*−04, One-sample *t*-test, *p* < 0.00001). HR also showed a positive correlation with absolute score indicating that HR increases as the game imbalances in favor of one side (One-sample *t*-test of the regression coefficients obtained from each game, *p* < 0.005).

On summary, HR increased throughout the phases of the game, when less time is available and when score became unbalanced. These three variables are correlated, as shown in Figures 1A,B, and our data could not distinguish how these strongly correlated factors differentially contribute to HR since a multiple regression to these factors was highly unstable. However, the non-stationary nature of HR throughout the game must be carefully taken into account for a robust analysis directed to our main goal: understanding how transient events of the game (occurrence of plans, calculation, blunders) relate to HR fluctuations.

### Transient modifications of HR

We analyzed HR dynamics in a 10 s time-window centered in the execution of the move. We used two procedures to assure that this analysis was not biased by non-stationarities of the data reported in the previous section. First, each move was normalized to its baseline, hence compensating for linear global trends. Second, to further compensate non-linear global trends, we performed a matching procedure (see “Materials and Methods”).

#### HR dynamics in blunders and correct moves

**Figure 2:**
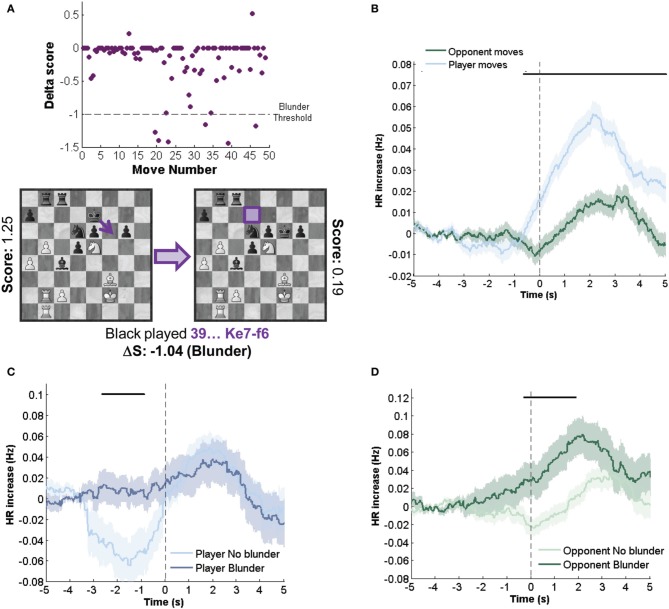
**HR indexes objective errors and this depends on which player did the move. (A)** Upper panel: Delta Score (Δ*S*) variation with move number for a typical game (Δ*S* ≤ −1 is a blunder). Lower panel, an example of two chess positions before and after a blunder or error move. Note that black's move allows white to fork the king and rook with the knight. **(B)** Temporal course of HR dynamics on own and opponent not error moves locked to move onset. **(C,D)** Dynamics of a player **(C)** and opponent **(D)** move for blunders and correct moves. For blunder versus non-blunder moves, matching conditions minimized differences in player AT and S. Black bars indicate intervals where differences between temporal series were significant (*p* < 0.05). Mean ± SEM across all games.

First, we simply compared HR dynamics on player versus opponent moves, excluding all blunders (Figures 2A,B). This comparison showed a significant difference between player and opponent moves from −0.5 to 5 s after the move (*p* < 0.05, see “Materials and Methods”).

HR responses locked to an opponent move were virtually flat until about 500 ms before the move. This anticipated response is not unexpected since a player can predict the timing of an opponent estimating the Hazard rate (Janssen and Shadlen, 2005) and also from gestures by the opponent. After the opponent move there is a change in HR with an effect size which peaks at about 0.02 Hz above the basal HR.

HR responses locked to the player's own moves showed a qualitatively different pattern. First there was a decrease in HR which started almost 3 s before the move. This trend did not reach significance. HR then ramped before the move reaching an almost threefold increase in modulation compared to opponent moves, peaking at 0.06 Hz modulation of baseline activity.

Interestingly, the early deep prior to the move was the most sensitive to the contrast between blunders (Δ*S*≤ −1) and correct moves Δ*S* ≥ −0.3) (Figure 2C). This modulation was virtually absent when the player blunders and was more pronounced when observing solely those trials in which there was not errors, but where errors were likely. We emphasize the difference between the light-blue-trace of Figure 2B (all own-moves, no blunders) with the light-blue-trace of Figure 2C (own moves, no blunders, but other variables matched to moves where blunders are made). The latter corresponded to a subset of the game, typically not including the opening, with less time available, where errors are more frequent, but selecting those cases in which errors were not made. In this specific filter which focused on difficult moments of the game, the early deep in HR was hence indicative, on average, of the quality of the move. In fact, this comparison (Figure 2C) revealed that only the −2.5 to −1 s interval showed a significant difference in HR for blunders versus non-blunders moves.

The comparison of blunders and non-blunders in opponent moves showed a very different pattern. Opponent blunders induced a higher HR increase than opponent non-blunder moves which was significant in the −0.25 to 2 s interval, almost entirely after the opponent move (Figure 2D).

#### HR dynamics in retrospectively reported cognitive events

**Figure 3:**
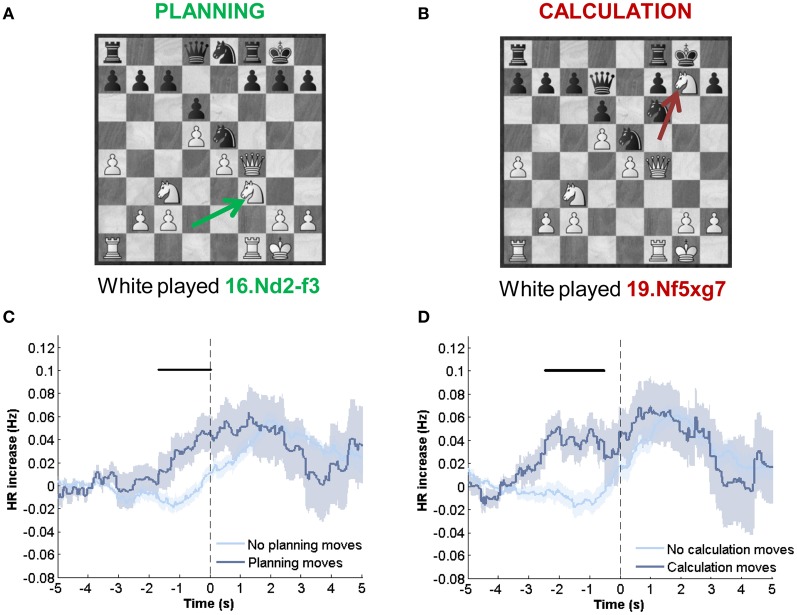
**HR indexes subjective and retrospectively highlighted events. (A)** Example of a planning move. In the position, white player just made a planning move (retrospectively highlighted after the conclusion of the game). White player plan was to initiate an attack in the king's flank, starting with the transference of one of his/her knight to f5 square. Nd2-f3 (indicated with an arrow) was the first move of this plan. **(B)** Calculation move. Later in the same game, other move was highlighted as calculation (19.Nf5-g7). In this case, player was thinking about specific variations (main variation: 19.Nf5-g7 Kg8-g7, 20.Qf4-f6+ Kg7-g8, (a) 21.Rf1-f5 or (b) 21.Nc3-b5 or (c) 21.Ra1-a3). **(C)** HR dynamics comparison of planning moves and non-planning moves matched by other variables. **(D)** HR dynamics comparison of calculation moves and non-calculation moves matched by other variables. All moves were no blunders and matching conditions minimized differences in player AT and S. Those moves highlighted as both planning and calculation were not considered. Black bars indicate intervals where differences between temporal series were significant (*p* < 0.05). Mean ± SEM across all games.

After the game, players reported in which moves they were engaged in the elaboration of a strategic plan or in calculation of variations. Players also reported their perceived errors and the moves that they ranked as especially good moves but we did not have sufficient records of these events to perform significant statistical analysis. For planning and calculation moves (Figures 3A,B) we found an increase in HR anticipating the move, compared to their respective matches. For planning moves, significant differences were found from −1.5 to 0 s (Figure 3C). For calculation moves, differences were found from −2.5 to −0.5 s (Figure 3D). Thus, both planning and calculation induced higher HR levels before the move.

### Classification analysis

Previous results pointed differences in mean HR for specific objective or subjectively labeled moves. In the vast majority of studies, this has been used as a statistical indicator that a variable (HR) indexes or informs about a state (blunder, calculation). Here we went beyond average difference estimators, observing the capacity of HR data to predict in a trial by trial basis, move observables such as its quality and the thought processes involved. It is clear that this analysis pushes the data to its limit since the HR is an intrinsically noisy signal. Specifically, we quantified the degree of separability of these temporal series training a linear decoder, using the SVM algorithm (Cristianini and Shawe-Taylor, 2000). We ran the classifier on data which were clearly before (3–1 s before the move) and after (1–3 s after the move) in the four comparisons described above. Classification was significant for all comparisons (Table 1), yielding classification values which were around 60% and revealing a trend which was consistent with the average data. This means that when using a leave-out procedure, where a subset of the data is used for training and another subset for testing, the performance of the classifier is correct in 60% of the test trials. Since this is a binary classification, chance level is at 50%. Calculation and planning were decoded better using data before the move and opponent blunders with data after the opponent move. The weakest classification was achieved for the player owns blunders, were classification was almost at chance. This is an interesting observation showing that fluctuations due to own blunders elicit a broad variety of changes and hence are less amenable to be captured by a linear classifier.

**Table 1:** **Linear classifier results**.

**Move type**	**Before (%)**	**After (%)**	***N* (targets)**
Player blunders	54.94 ± 3.35	46.09 ± 1.45	24
Opponent blunders	56.50 ± 2.03	62.46 ± 1.55	34
Planning	56.87 ± 2.92	55.56 ± 3.23	15
Calculation	78.18 ± 2.96	58.14 ± 2.63	17

## Discussion

Our work shows that beyond known modulations of body signals in decision making (Bradley, 2009; Jennings et al., 2009; Taelman et al., 2011), HR can signal relevant cognitive episodes including objective events such as the correctness of choice and subjective events tagged by retrospective reports such as engaging in a plan or in calculation relevant for multi-step cognition (Anderson and Lebiere, 1998). Thus, even if HR constitutes a relatively broad marker integrating a myriad of physiological variables, its dynamic was rich enough to reveal relevant episodes of inner thought.

The seminal work of Adriaan de Groot used chess as a vehicle to understand thought (De Groot, 1965). This work relied on introspection, using the methodology of thinking aloud as the main vehicle to identify episodes of thought. Here we showed that the HR signal carries information capable of indexing these episodes: increasing before player own blunders, planning and calculation moves, and reacting to opponent errors.

Previous studies have investigated how HR varies in longer chess games, consistently finding an increase in HR throughout the game (Pfleger et al., 1980; Hollinsky et al., 1997; Troubat et al., 2009). We replicated this effect suggesting a universality of this phenomenon observed in different time-scales, experimental setups and specific analytic measures of HR.

Our aim here was to understand how on top of this global trend, the HR signal is modulated by specific episodes which relate to strategies, calculations, and the outcomes of decisions made during the game.

First, we observed a very different dynamics when HR variations were locked to a player or to the opponent move. Changes in dynamics after the move could have two different origins which here we cannot disambiguate. First it could be simply the effect of the motor action. Second, and more interesting, it is possible that mechanisms of evaluation of one's own action engage a larger increase in HR than the evaluation of the opponent action.

For player moves, the significant differences between errors and good moves were found in the region corresponding to the recorded player's turn to play. We found a very robust marker in the HR signal which anticipated a correct decision, which involved a transient decrease in the HR prior to the move. This was apparent in all the data and much more strikingly when considering moves which were paired to errors (in the same parameters and state of the game) but in which the error was not made. This is consistent with the observation of HR decreases before making a good decision, only observed for risky choices and good performers on IGT (Crone et al., 2004). It is important to keep in mind that errors are typically made in tense situations of the game (time trouble, unbalanced score). In a simple and naive model in which HR indexes the load of rational thought (consistent with our observation of increased HR with calculation and planning) this deep could be understood as a comparable absence of rational thought. Following this logic and only as a driving hypothesis we suggest that in line with several behavioral observations, in such complicated situations, it might be better to follow hunches than rational and deliberate thought (Dijksterhuis et al., 2006). This hypothesis is consistent with the observations of a HR deceleration immediately before an action which has been related to inhibition of other actions and preparation for the imminent stimulus (Jennings and Van Der Molen, 2002).

Finally, HR was also altered by cognitive processes related with problem solving, as planning (setting a goal) and calculation (analysis of specific candidate moves and their variation). Planning and calculation moves (both are player moves) showed a similar pattern on HR compared to other matched moves: they had an HR increase before the signaled move. A particularly motivating challenge for future research is to understand the causal relation of this observation. As argued above, it may be that the load of rational thought induces transient increases of HR. Alternatively, pushing farther the SM hypothesis it is possible that SM do not only assist choice in overt actions but also signal internal episodes of a mental program (Duncan, 2010; Zylberberg et al., 2011). In other words, it is possible that the action by which a player makes a pause in the game, changes a plan, engages on deep calculation is flagged by internal somatic variables, like HR.

All the previous discussion was drawn analyzing how a factor affects the mean of a distribution. This is the most classic analysis by which inferences are drawn from significant global tendencies of the data. In HR data it seems difficult to go beyond these estimates because of the intrinsic high noise of the signal.

Here we made an effort in this direction, zooming in to single-trial analysis to inquire which factors produce reliable changes which serve to decode states from the data. We used a linear classifier procedure which essentially relies on a bisection of the data by a plane. This method effectively decodes when the factor produces a consistent (albeit noisy) perturbation in the data. If instead, a factor produces a myriad of different changes which when summed together produce a change in the mean, the decoder is not effective. Hence, one can see this analysis as a way to inquire the consistency of an effect. Our data showed a reliable classification for three of the four factors: planning, calculation, and opponent blunders. The most effective decoding was for calculation, when relying on data before the move, which reached levels above 75% which are considerably high for HR data which, as expected, has multiple sources of noise. Instead, the classification for own blunders was very modest, almost at chance levels. This is in fact a very robust result as even varying the parameters of the classifier; these numbers remain close to chance. We suggest that this data reflect that compared to planning, calculating or to the observation of an error of the opponent, one's own blunder may reflect many different internal processes which, in turn, affect the heart in different manner. Interestingly, decoding was effective in introspective variables which could not be measured without explicit reports.

### Conflict of interest statement

The authors declare that the research was conducted in the absence of any commercial or financial relationships that could be construed as a potential conflict of interest.

## Appendix

### Post-game questionnaire (cuestionario post-partida)

Player name (Nombre del jugador): _____________________________ Game (Partida): _________________________________

Round (Ronda): ________________________________ Game Result (Resultado): _______________________________________

Opening theorical knowledge (YES/NO) (Conocimiento teórico de la apertura (SI/NO)): ___________________________________

Last move known (Hasta qué jugada): ____________________________________________________________________________

Planning moments (Momentos de generación de plan): ______________________________ (please, write the move corresponding to the plan start) (escribir la jugada propia correspondiente a su inicio).

Game style (Estilo de juego de la partida): _____________________

Does the style agree with your own style? (YES/NO) (¿Acorde al propio? (SI/NO)): _________________________________________

Tactical calculation moments (Momentos de cálculo táctico): ___________________ ______________________________________

**Highlighted moves** (Jugadas destacadas):

- *Sacrifices* (Sacrificios): _______________________________________________________________________________________

**Errors** (Errores):

1. **Own errors** (Propios)
  - Unconscious and opponent took advantage of it (concientes y aprovechados por el rival): ___________________________
  - Conscious (after its execution) and opponent took advantage of it (concientes (luego de su realización) y aprovechados por el rival): _____________________________________________________________________________________________
  - Conscious (after its execution) and opponent did NOT take advantage of it (concientes (luego de su realización) y NO aprovechados por el rival): _____________________________________________________________________________
2. **Opponent errors**(Del adversario)

    You took advantage of it (Aprovechados): _________________________________________________________________________

    You did NOT take advantage of it (NO aprovechados): _______________________________________________________________

**Good moves** (Buenas jugadas):

    Own moves (Propias): ____________________________________________________________________________________

    Opponent moves (Del Adversario): __________________________________________________________________________

## References

[B1] Anderson J. R. Lebiere C. (1998). The Atomic Components of Thought. Mahwah, NJ: Lawrence Erlbaum Associates

[B2] Ariely D. (2008). Predictably irrational. The Hidden Forces That Shape Our Decisions. NewYork, NY: Harper/HarperCollins Publishers

[B3] Bechara A. Damasio A. (2005). The somatic marker hypothesis: a neural theory of economic decision. Games Econ. Behav. 52, 336–372

[B4] Bechara A. Damasio A. R. Damasio H. Anderson S. W. (1994). Insensitivity to future consequences following damage to human prefrontal cortex. Cognition 50, 7–15 8039375 10.1016/0010-0277(94)90018-3

[B5] Bechara A. Damasio H. Tranel D. Damasio A. R. (1997). Deciding advantageously before knowing the advantageous strategy. Science 275, 1293–1295 10.1016/j.neuropsychologia.2006.01.014 9036851

[B6] Bradley M. M. (2009). Natural selective attention: orienting and emotion. Psychophysiology 46, 1–11 10.1111/j.1469-8986.2008.00702.x 18778317 PMC3645482

[B7] Cristianini N. Shawe-Taylor J. (2000). An Introduction to Support Vector Machines and Other Kernel-based Learning Methods. Cambridge: Cambridge University Press

[B8] Crone E. A. Jennings J. R. Van Der Molen M. W. (2003). Sensitivity to interference and response contingencies in attention-deficit/hyperactivity disorder. J. Child Psychol. Psychiatry 44, 214–226 10.1111/1469-7610.00115 12587858

[B9] Crone E. A. Somsen R. J. Van Beek B. Van Der Molen M. W. (2004). Heart rate and skin conductance analysis of antecendents and consequences of decision making. Psychophysiology 41, 531–540 10.1111/j.1469-8986.2004.00197.x 15189476

[B10] Damasio A. R. (1994). Descartes' Error: Emotion, Reason, and the Human Brain. New York, NY: Grosset/Putnam

[B11] De Groot A. (1965). Thought and Choice in Chess. The Hague: Mounton

[B12] Dijksterhuis A. Bos M. W. Nordgren L. F. Van Baaren R. B. (2006). On making the right choice: the deliberation-without-attention effect. Science 311, 1005–1007 10.1126/science.1121629 16484496

[B13] Drucaroff L. J. Kievit R. Guinjoan S. M. Gerschcovich E. R. Cerquetti D. Leiguarda R. (2011). Higher autonomic activation predicts better performance in iowa gambling task. Cogn. Behav. Neurol. 24, 93–98 10.1097/WNN.0b013e3182239308 21677576

[B14] Duncan J. (2010). The multiple-demand (MD) system of the primate brain: mental programs for intelligent behaviour. Trends Cogn. Sci. 14, 172–179 10.1016/j.tics.2010.01.004 20171926

[B15] Dunn B. D. Dalgleish T. Lawrence A. D. (2006). The somatic marker hypothesis: a critical evaluation. Neurosci. Biobehav. Rev. 30, 239–271 10.1016/j.neubiorev.2005.07.001 16197997

[B16] Elo A. (1978). The Rating of Chess Players, Past and Present. London: Batsford

[B17] Hollinsky C. Maresch G. Hiller M. Kohlberger P. Bieglmayer C. (1997). Beeinfluß t körperliche Fitneß die Leistungsfähigkeit von Ranglistenschachspielern. Ö J. Sportmed. 27, 51–59

[B18] Janssen P. Shadlen M. N. (2005). A representation of the hazard rate of elapsed time in macaque area LIP. Nat. Neurosci. 8, 234–241 10.1038/nn1386 15657597

[B19] Jennings J. R. Van Der Molen M. W. (2002). Cardiac timing and the central regulation of action. Psychol. Res. 66, 337–349 10.1007/s00426-002-0106-5 12466930

[B20] Jennings J. R. Van Der Molen M. W. Tanase C. (2009). Preparing hearts and minds: cardiac slowing and a cortical inhibitory network. Psychophysiology 46, 1170–1178 10.1111/j.1469-8986.2009.00866.x 19572902

[B21] Kotov A. (1971).Think like a GrandMaster. London: B.T. Batsford Ltd

[B22] Maia T. V. McClelland J. L. (2004). A reexamination of the evidence for the somatic marker hypothesis: what participants really know in the Iowa gambling task. Proc. Natl. Acad. Sci. U.S.A. 101, 16075–16080 10.1073/pnas.0406666101 15501919 PMC528759

[B23] Pfleger H. Stocker K. Pabst H. Haralambie G. (1980). [Sports medical examination of top class chess players (author's transl)]. MMW Munch. Med. Wochenschr. 122, 1041–1044 6775202

[B24] Sigman M. Etchemendy P. Slezak D. F. Cecchi G. A. (2010). Response time distributions in rapid chess: a large-scale decision making experiment. Front. Neurosci. 4:60 10.3389/fnins.2010.00060 21031032 PMC2965049

[B25] Slezak D. F. Sigman M. (2011). Do not fear your opponent: suboptimal changes of a prevention strategy when facing stronger opponents. J. Exp. Psychol. 141, 527–538 10.1037/a0025761 22004170

[B26] Taelman J. Vandeput S. Vlemincx E. Spaepen A. Van Huffel S. (2011). Instantaneous changes in heart rate regulation due to mental load in simulated office work. Eur. J. Appl. Physiol. 111, 1497–1505 10.1007/s00421-010-1776-0 21188414

[B27] Tranel D. Bechara A. Damasio A. R. (1999). Decision making and the somatic marker hypothesis, in The New Cognitive Neurosciences, 2nd Edn, ed Gazzaniga M. S. (London: The MIT Press), 1047

[B28] Troubat N. Fargeas-Gluck M. A. Tulppo M. Dugue B. (2009). The stress of chess players as a model to study the effects of psychological stimuli on physiological responses: an example of substrate oxidation and heart rate variability in man. Eur. J. Appl. Physiol. 105, 343–349 10.1007/s00421-008-0908-2 18987876

[B29] Van Der Maas H. L. Wagenmakers E. J. (2005). A psychometric analysis of chess expertise. Am. J. Psychol. 118, 29–60 15822609

[B30] Zylberberg A. Dehaene S. Roelfsema P. R. Sigman M. (2011). The human Turing machine: a neural framework for mental programs. Trends Cogn. Sci. 15, 293–300 10.1016/j.tics.2011.05.007 21696998

